# Supplementary figures and images for: Towards a potent and rapidly reversible Dexmedetomidine-based general anesthetic
Source: PLoS One. 2023 Sep 26;18(9):e0291827. doi: 10.1371/journal.pone.0291827 (PMC10522005; doi:10.1371/journal.pone.0291827)

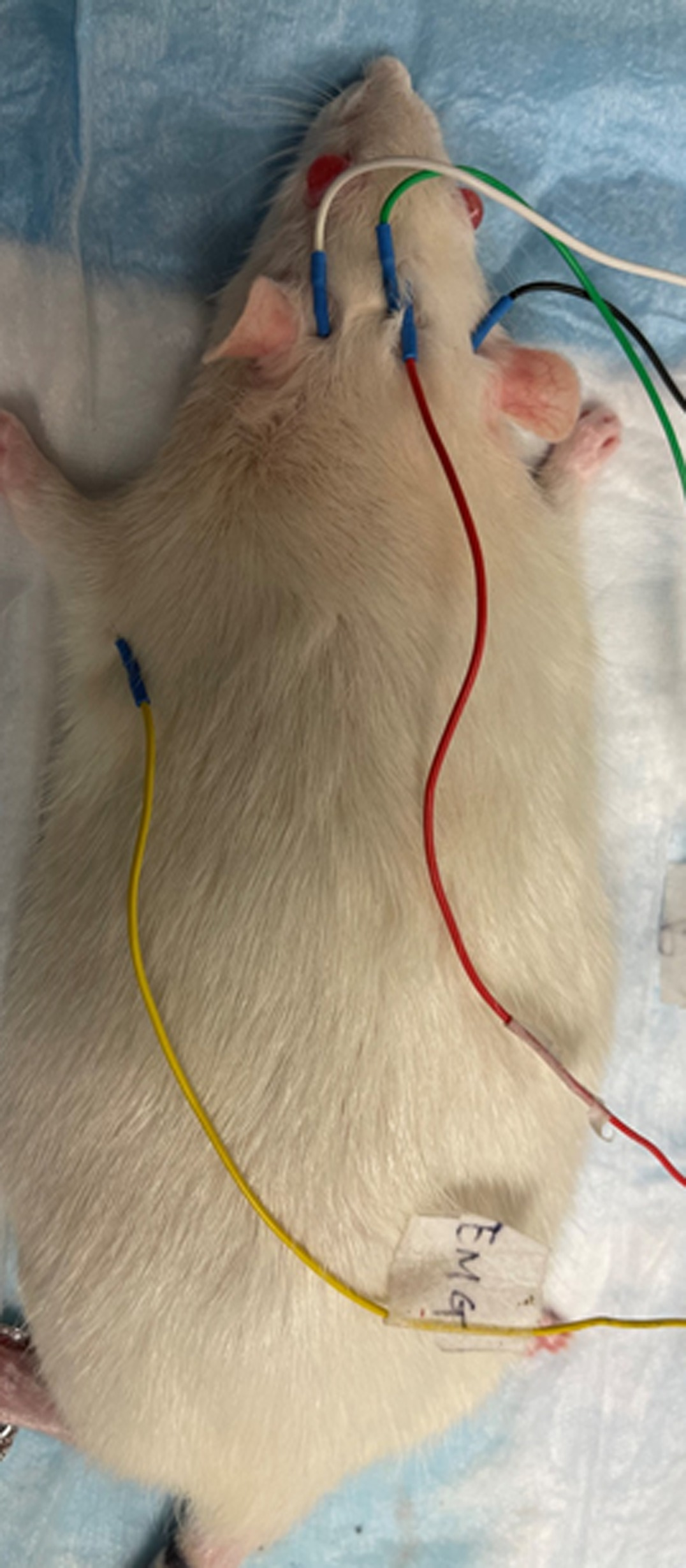

Supplement: S1 Fig — Two scalp electrodes were placed, as shown. We drew a line between the anterior edge of bilateral ears, between Bregma and Lambda. From the midpoint, one electrode was placed anteriorly perpendicular to the line and the other posteriorly perpendicular to it. Two EEG channels were recorded, the first one (red) from an electrode placed over the anterior portion of the brain, and a second electrode (green) placed over the posterior portion of the brain. The EMG lead (yellow) was obtained from an electrode placed over the left shoulder, all referenced to an electrode (white) placed near medial to the ears. A ground electrode (black) was placed on the opposite side of the reference lead. Signals recorded from the anterior lead were analyzed for global changes during anesthesia. (TIF) [file pone.0291827.s002.tif]

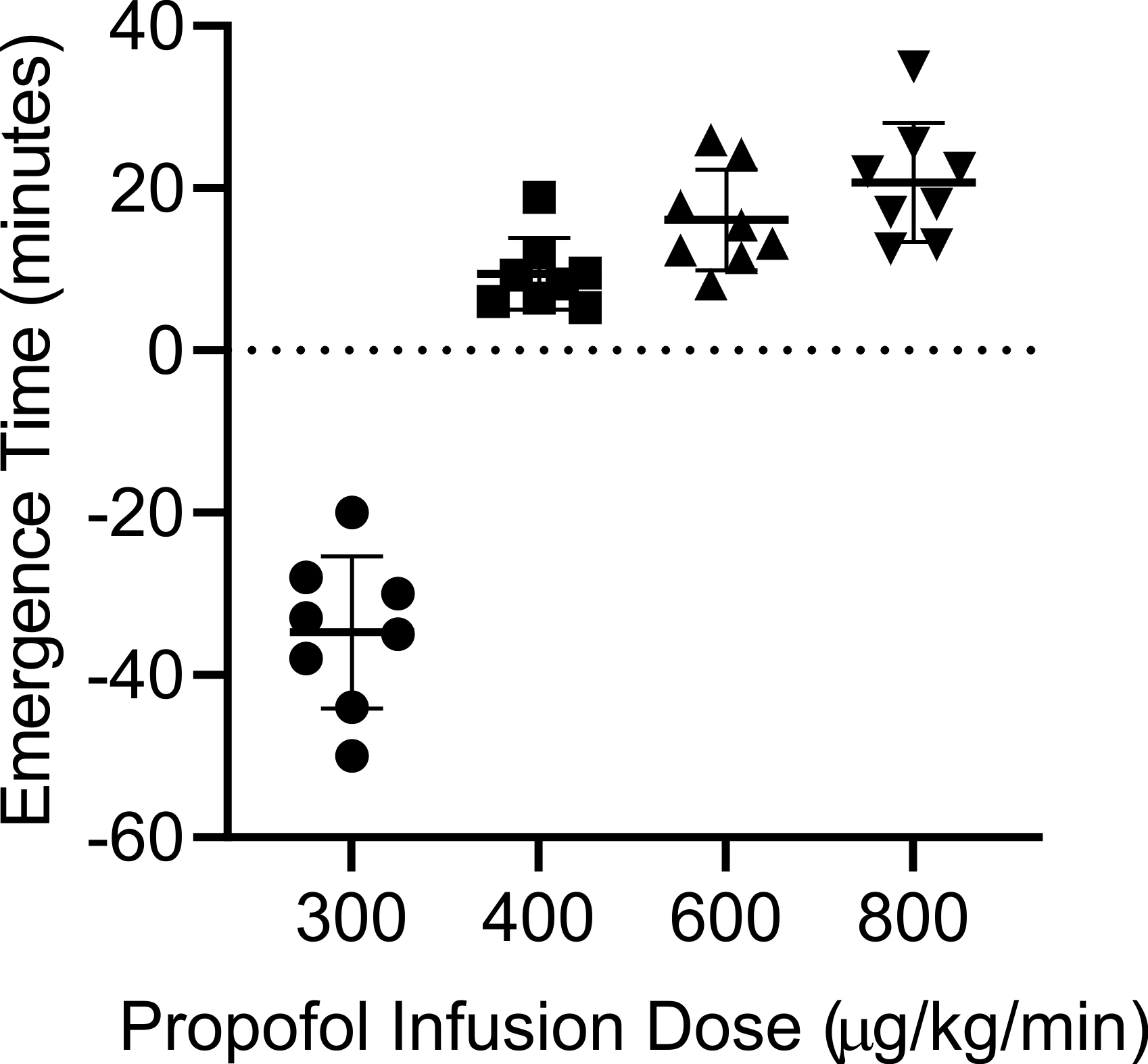

Supplement: S2 Fig — For this experiment a group of rats received a bolus of 5 mg/kg of propofol, applied in 5 minutes via a pump, after which they received a continuous infusion of propofol, at different concentrations for an additional 60 minutes. All rats remained unconscious for the 60-minute infusion if the infusion rates of propofol were kept at or above 400 μg/kg/min. In contrast, 300 μg/kg/min was not sufficient to keep the rats unconscious during the infusion. (TIF) [file pone.0291827.s003.tif]

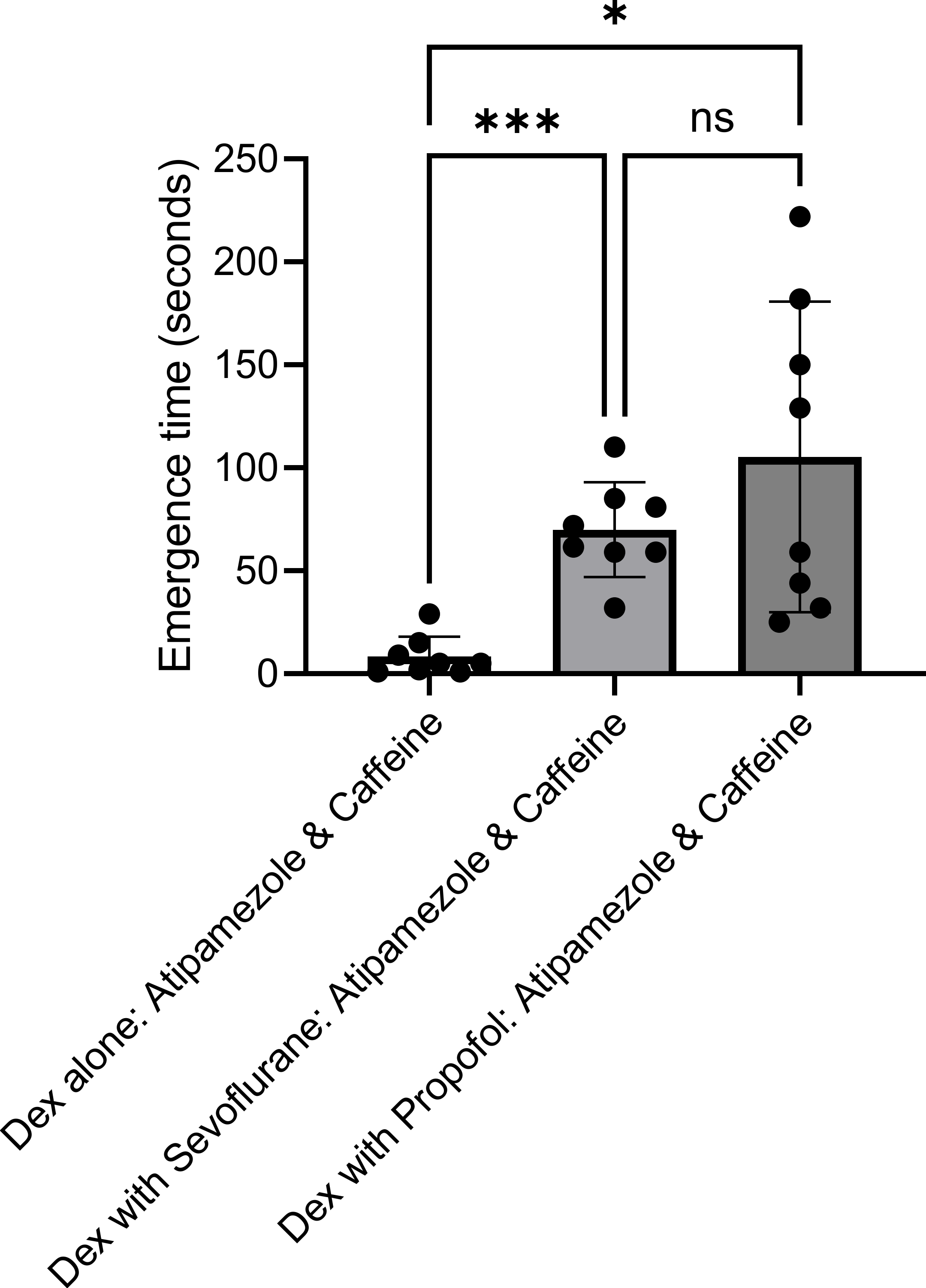

Supplement: S3 Fig — The same group of 8 rats were exposed to three sedation sessions, a week apart. At the end of each session the rats received a bolus injection of atipamezole (10 μg/kg) and caffeine (25 mg/kg). Rats were placed on their backs in a waking box, and the time for the rats to recover their righting reflex was recorded. Data (RORR Times in seconds): Dex alone—5, 9, 5, 1, 29, 1, 15, 2, Dex with Sevoflurane—110, 72, 81, 59, 62, 32, 85, 59, Dex with Propofol—222, 182, 150, 44, 32, 59, 129, 25. (TIF) [file pone.0291827.s004.tif]

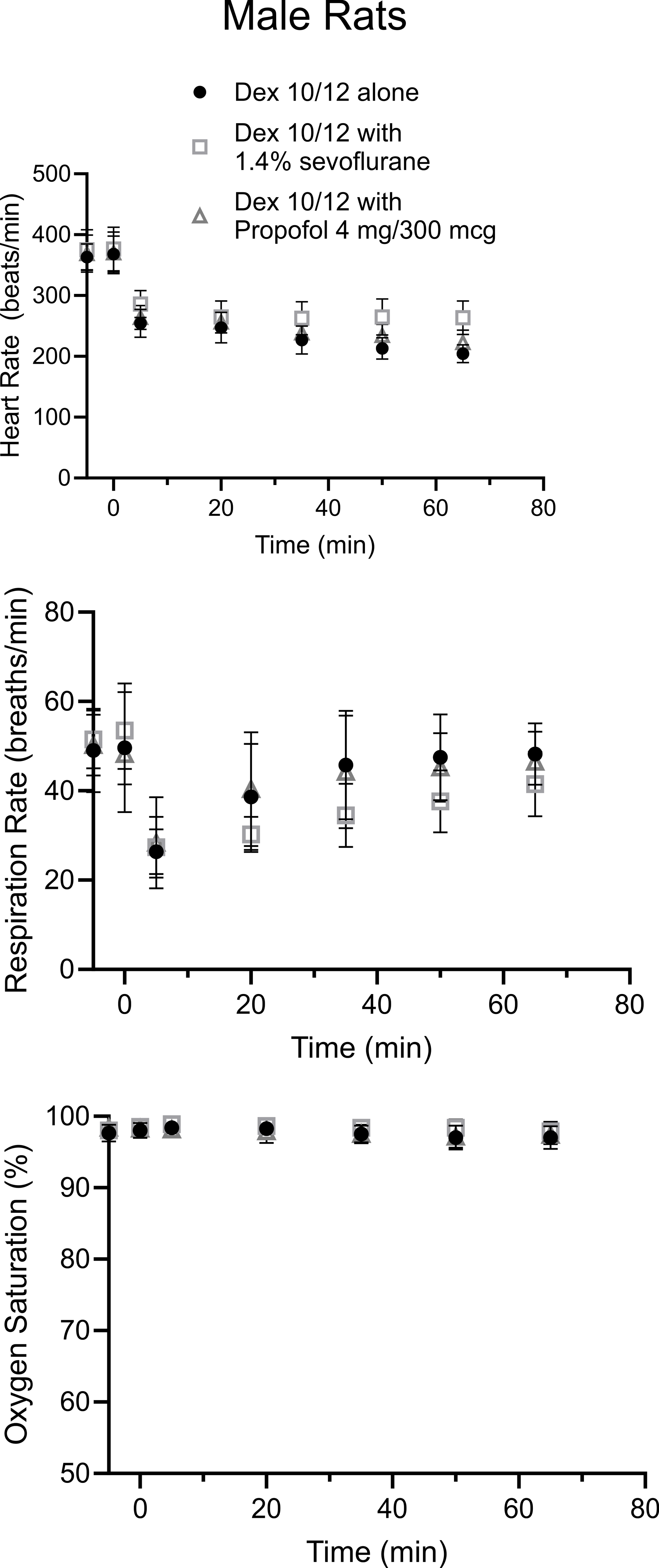

Supplement: S4 Fig — Dex or Dex supplemented with either propofol or sevoflurane was applied at time = 0. Vital signs were then measured every 15 minutes. Comparisons of HR at different times for Dex 10/12. Dex/sevoflurane and Dex/propofol were similar and are not presented. For this analysis a repeated measures two-way ANOVA was employed: 1.7% isoflurane vs. 1.1% isoflurane, p = ns: We compared the HR at 1.1% isoflurane to the rest of the time points. 1.1% isoflurane vs. End of Bolus, p <0.0001: 1.1% isoflurane vs. t = 15, p = 0.0001: 1.1% isoflurane vs. t = 30, p <0.0001: 1.1% isoflurane vs. t = 45, p < 0.0001: 1.1% isoflurane vs. t = 60, p <0.0001, Comparisons of RR: Only the following times were different. 1.1% isoflurane vs. End of Bolus, p < 0.0001: End of bolus vs t = 30, p = 0.0006: End of bolus vs t = 45, p = 0.0001: End of bolus vs t = 60, p < 0.0001: Comparisons of SpO2: No significant changes were observed. (TIF) [file pone.0291827.s005.tif]

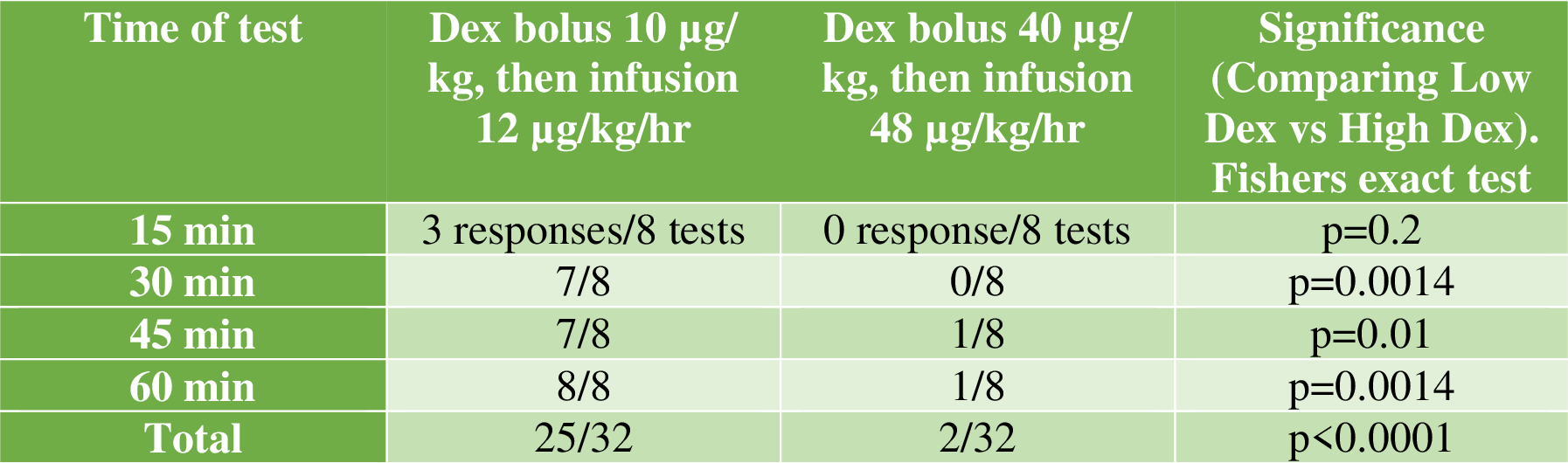

Supplement: S1 Table — A noxious stimulus was applied at different time points in an experiment. Responses were tabulated and are presented numerically in the table. Statistical difference is calculated with Fisher’s exact test. (TIF) [file pone.0291827.s006.tif]

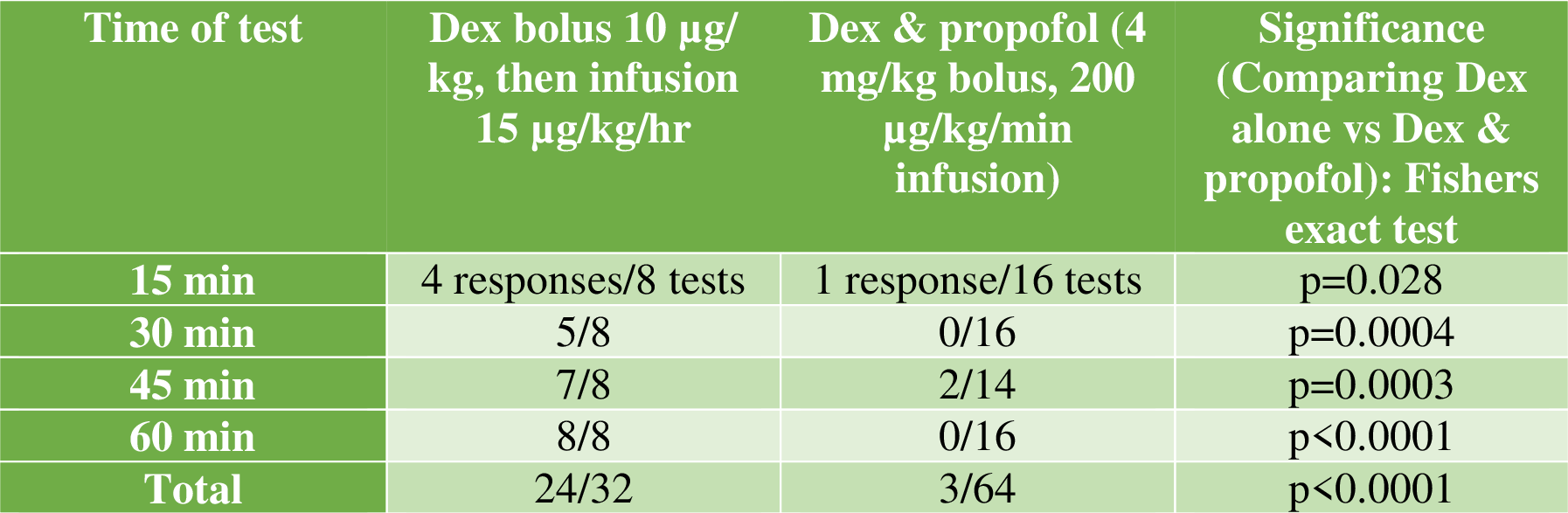

Supplement: S2 Table — A noxious stimulus was applied at different time points in an experiment. Responses were tabulated and are presented numerically in the table. Statistical difference is calculated with Fisher’s exact test. (TIF) [file pone.0291827.s007.tif]

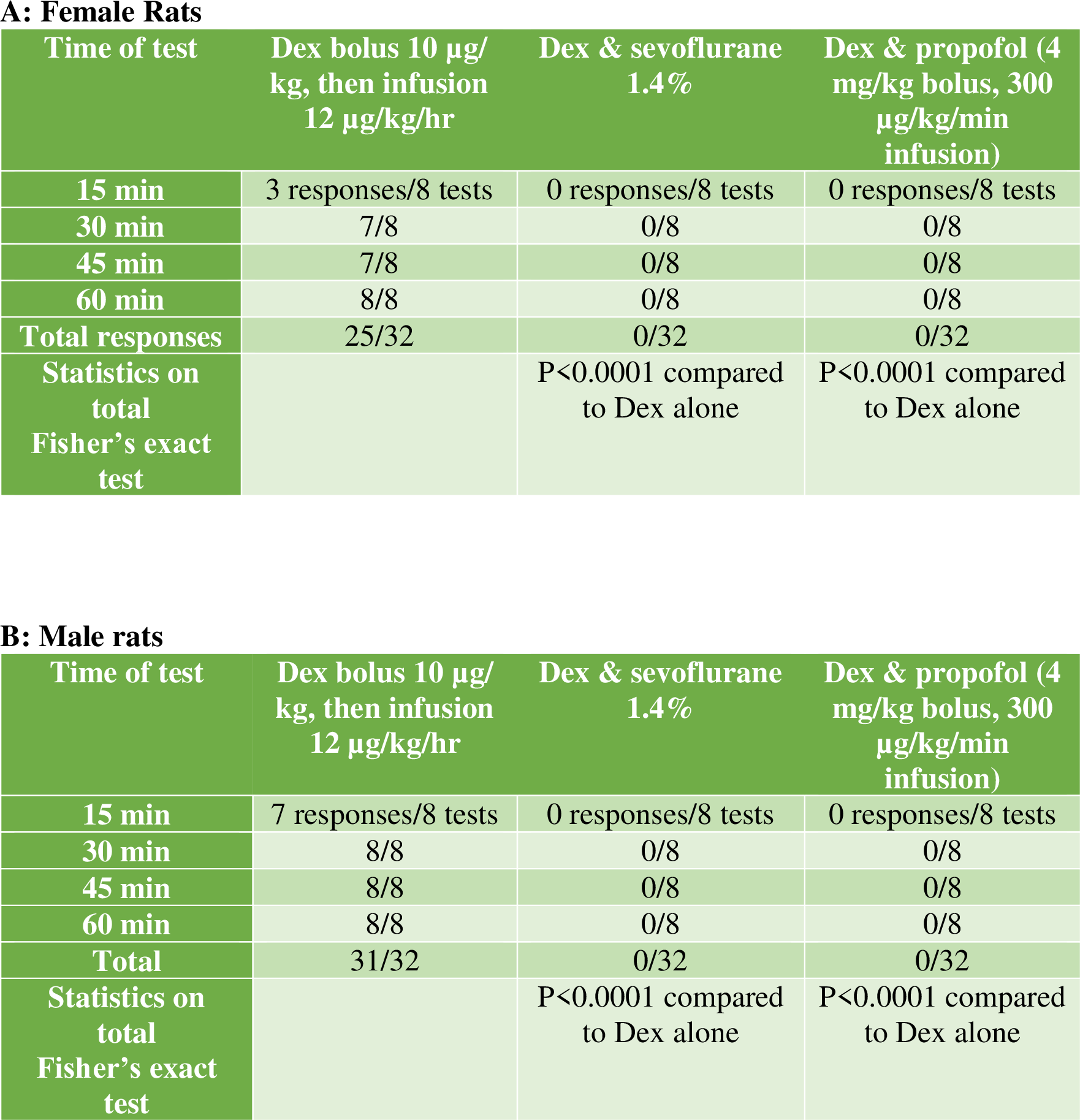

Supplement: S3 Table — A noxious stimulus was applied at different time points in an experiment. Responses were tabulated and are presented numerically in the table. Statistical difference is calculated with Fisher’s exact test. A, data from female rats. B, data from male rats. (TIF) [file pone.0291827.s008.tif]

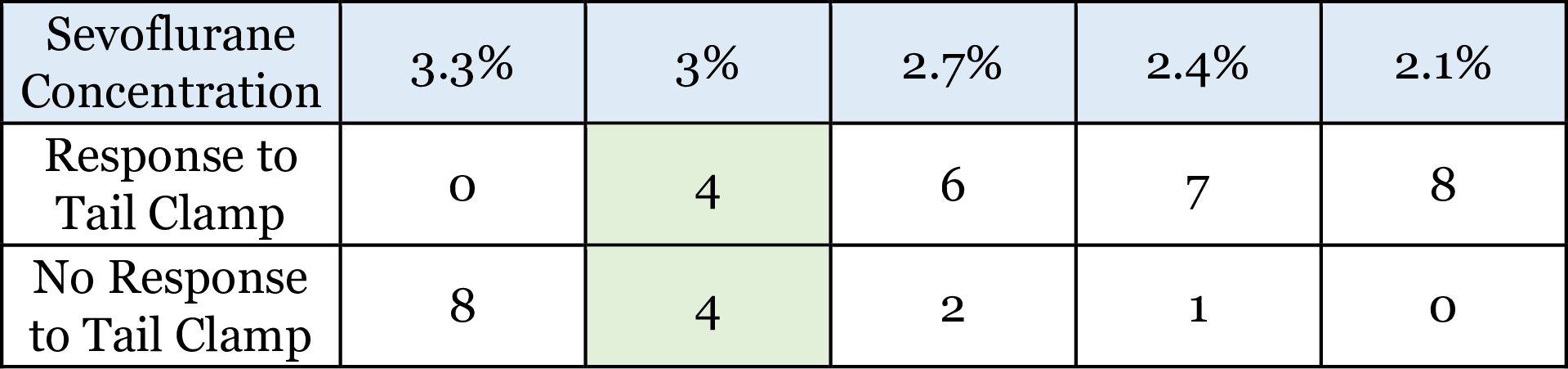

Supplement: S4 Table — Details about the stimulus are in the Methods. The green column represents ~1 MAC equivalence concentration or EC50. (TIF) [file pone.0291827.s009.tif]
