# Supplementary material for: Towards a potent and rapidly reversible Dexmedetomidine-based general anesthetic
Source: PLoS One. 2023 Sep 26;18(9):e0291827. doi: 10.1371/journal.pone.0291827 (PMC10522005; doi:10.1371/journal.pone.0291827)
Supplement: Attachment — Submitted filename: Point by Point Rebuttal Letter.8.18.23.docx [file pone.0291827.s011.docx]

PONE-D-23-07486R2

**Towards A Potent and Rapidly Reversible Dexmedetomidine-Based General Anesthetic**

August 18, 2023

Dear Dr Fiorelli,

We would like to thank the reviewers for their diligence. Multiple changes were made in response to the reviews to address the comments.

This letter includes a point-by-point response to each of the comments made by the reviewers and the editor. The reviewer’s comments are in bold italics followed immediately by our reply in regular font. The line numbers in the reply to the reviewers correspond to the line numbers in the version with “Tracked Changes – Simple Markup.”

***Reviewer #1: In Manuscript PONE-D-23-07486_R1, Xie and colleagues have revised their prior manuscript as per the reviewers’ comments. Overall, I appreciate the amount of work and new experiments performed for this revision. By concentrating only one two general anesthetic agents, sevoflurane (sevo) and propofol (prop), the authors have made the paper more focused and thus*** *their* ***conclusions are more easily appreciated. However, there are still some issues that need to be addressed (without more experiments) as discussed below.

1. The introduction is much improved in flow and focus. It is now clear that the authors are attempting to extend prior research on this topic. Previously, others have shown that co-administration of dexmedetomidine (dex) can reduce the MAC (or concentration) of sevo or propofol necessary to achieve an anesthetic state. Namely,*** ***the authors are attempting to discern if higher doses of dex co-administered with sevo, or prop can achieve an anesthetic state while using a sub-anesthetic dose of the same.

By describing why high dose dex alone as an anesthetic agent has failed in the past (e.g., slow emergence, lack of good reversal agents, vital sign effects), it becomes more apparent why further work is needed. However, I still struggle with the idea that the combination of dex with other agents can lead to dex being the “primary” agent. For example,*** ***prior work has shown that dex can reduce the MAC of another agent. In this work, the authors have also shown that dex can reduce the MAC of another agent, possibly reducing the second agent (sevo or prop) to a concentration/dose that is now considered sub-anesthetic. In other words, the authors appear to not be doing something unique but rather extend prior work to see if the MAC lowering effect of dex can be extended to reduce MAC further by using a higher dose of dex than that studied previously.***

Thank you for your thoughtful comments. The reviewer correctly pointed out that “**The authors are attempting to discern if higher doses of Dex co-administered with sevo, or prop can achieve an anesthetic state while using a sub-anesthetic dose of the same.**” The reviewer is also correct that “**prior work has shown that dex can reduce the MAC of another agent.**” Most of the previous studies using Dex to reduce MAC levels were done in humans (Ref 61,63,64). Here is a summary of the previous studies in question: Dex was used an adjunct agent at the doses ranging from 0.3-0.7 mcg/kg/hr which reduced the MAC concentration of sevoflurane. In those studies, the modest dose of Dex that was employed, reduced the MAC of sevoflurane by 20-30%. In one pediatric study, low dose Dex (0.2 mcg/kg/hr), reduced the required sevoflurane dose by 60%. However, these patients also received a caudal block after induction for the procedure, thus obviating the need to provide analgesia (Ref 62). In another study with isoflurane, higher doses of Dex (up to 2.85 mcg/kg/hr) were used in human volunteers. The MAC of isoflurane was reduced by 50%. However, the recovery time was very long (up to 4-6 hours) in those subjects (1, see reference below; We did not cite this article because we focused on sevoflurane and propofol in the MS). In a case report with three patients, higher doses of Dex (5-10 mcg/kg/hr) alone were used in conjunction with local anesthetics (ref 60) for surgery. Dex at these high doses, which were like the Dex dose (12 mcg/kg/hr) used in rats in our study, produced sufficient anesthesia for the procedures without significant hemodynamic compromise in the three patients. Again, their recovery time was about 2 hrs or longer.

Studies of high dose Dex have been hindered in the adult human population for two reasons. First, when Dex is used in the adult population the dosages are typically low. It is employed at dosages in the range 0.2 - 0.7 mcg/kg/hr. These low dosages already produce significant hemodynamic effects. The worry is that going to higher dosages will exacerbate the hemodynamic effects and this concern prevents higher doses being employed in adults. Second, as you can already see from the brief review of the literature that was presented above, higher doses of Dex produce enormously long emergence times in humans. Hours, to many hours is simply not acceptable. No one will use high dose Dex, even if it is demonstrably safer. It’s just not practical.

In rats, our studies show that the hemodynamic effects of Dex saturate at low concentrations. High dose Dex does not produce additional hemodynamic changes compared to low dose Dex. Most importantly, using our reversal agent, we change the emergence times from hours to seconds. Our results suggest that high dose Dex can be used as the primary agent safely and without tying up recovery facilities for hours. We believe that these results may change the way that clinicians look at Dex.

We agree with the reviewer that on one level we could interpret our study as an extension of some of the above previous work by using a higher dose of Dex to permit a further reduction of the required doses of sevoflurane or propofol. However, we would argue instead that our focus driving this work was not so much the reduction of the required quantities of other common anesthetizing agents using Dex as an “adjunct”, but rather the use of minimal amounts of other agents to allow Dex to function as an effective general anesthetic. On the surface, this characterization appears semantically tautological- much like the distinction between characterizing a glass as “half empty” vs “half full”. We would like to appeal to the very different philosophical and perspectival orientations that underlie the use of one description vs the other. Without an effective reversal for Dex, the use of higher doses of Dex will not be feasible clinically. The use of Dex will be limited at 0.2-0.7 mcg/kg/hr which will not produce unconsciousness in humans. It will be only used as the less potent agent of two agents regardless the combinations. In pediatric sedation, Dex is commonly used at 2-3 mcg/kg/hr. At this dose, most patients are deeply sedated or unconscious. At these doses of Dex, high doses of sevo or propofol are not needed. However, this high dose of Dex prolongs recovery if there is no safe and effective reversal. The unique nature of our study is that we can create a reversible anesthetic combination with more selective targets. We believe the higher the dose of sevoflurane or propofol the less selective they are. In this study, we cannot confirm whether higher dose of Dex and lower dose of sevo or propofol are safer in the elderly or less neurotoxic in neonates. We only confirm this approach is feasible and we will assess these combinations to determine their safety, efficiency and neurotoxic profiles in humans and animals in the future.

The reviewer pointed out that any agent could in principle be considered “primary” in our combinations because we require both Dex and the second agent to maintain robust general anesthesia. To justify our characterization, consider the analogy to how one assigns which component in a liquid/liquid solution is the “solvent” (since one could argue that either liquid could be considered a solvent). Conventionally this assignment is made based on the component that is present in greater chemical quantity. Similarly, we call Dex the “primary” agent in our anesthetic combinations because the Dex was present in greater “anesthetizing quantity” than the other agents with which it was paired. By this we mean that while Dex alone at the doses we used was sufficient to keep all rats unconscious for the duration of infusion, the second agents (propofol or sevoflurane) at the doses we used were unable *by themselves* to maintain unconsciousness and suppress righting in the rats. In addition, Dex was the drug we reversed. The doses of sevo or propofol were so low that no reversal for sevo or propofol was needed in these combinations.

As we extend our findings into human subjects, we will seek to establish an effective and safe dose in humans which is comparable to the doses of Dex in this animal study. We might start, for example, at 0.7-1 mcg/kg/hr. Pediatric sedation has employed doses up to 3 mc/kg/hr safely. Very likely the sweet spot will be somewhere in between. Armed with an effective reversal, if it proves to be effective, we will be empowered to evaluate Dex at doses higher than those we currently use when employing Dex as an adjunctive agent.

Finally, we defined “low dose” dex as 10 mcg/kg bolus and 12 mcg/kg/hr infusion vs “high dose” as 40 mcg/kg and 48 mcg/kg/hr based on the responses to tail clamp stimuli engendered. Dex at 10 mcg/kg and 12 mcg/kg/hr is considered a very high dose in human despite a case report (Ref 62) documenting comparable doses. In rat studies this dose is on the low end. With this low dose, all rats lost their righting reflex and remained unconscious, but not be able to tolerate any noxious stimuli, including IV insertion. In the study of amphetamine and Dex (ref. 77), those authors used a 50 mcg/kg bolus. It is used at even higher doses in veterinary medicine. We attempted to use the lowest dose of Dex which caused a loss of righting reflex to combine with other subanesthetic doses of sevo or propofol to produce reversable general anesthesia.

We have included these points in the discussion section (please see lines 504-531).

***2. The data reported in this study strongly suggests that the effect of dex to reduce MAC can be seen at higher doses of dex. The study then is extended to demonstrate that the effects of dex/sevo or dex/prop in a rat model of tail clamping (used to simulate surgical stimulation) can be extended to a sham surgery protocol. This protocol was included to assess if the behaviors seen in the rat model are similar to a surgical protocol. As noted by the authors, they could only measure changes in vital signs as a surrogate for anesthetic effects, but the data appears to support their hypothesis.***

Thank you for your understanding.

***3. One drawback to the use of dex at higher doses is the need for a reversal agent. As there is no reversal agent approved for humans use, the authors attempted to reverse dex with atipamezole and caffeine (as done in their prior published work). It is clear that this combination of reversal agents works.***

Thank you very much.

***4. Lastly, in order to determine if the newer combinations of dex with either sevo or propofol produce amnesia, the authors opted to study these anesthetic combinations with EEG analysis. There is a lot of data and I appreciate that the authors feel it is important to include this data due to concerns that potential reviewers may not agree with their findings without evidence of amnesia.***

Thank you for your understanding.

***5. Therefore, the current revision is much improved and more focused in design, but not more focused in the writing. The paper contains large sections of discussions of early data. For example, Page 90 Paragraph 2 is very long to simply make the point that dex suppressed responses to noxious stimuli. These discussions and tables (Table 1-3) are not necessary or should be moved to the supplemental data area. The paper is still too long for the conclusions reached and such detail makes reading the paper difficult.***

We revised the discussions as instructed by the reviewer. The paragraph 2 in page 90 (previous tracked version) was shortened. Tables 1-3 were moved to supplemental data area. We only described the tail clamp information in the result section.

***6. If the authors feel the need to retain the section of EEG analysis, it needs to be shortened. Does all of the detail need to be included?***

We feel the EEG analysis is critical in establishing some of the conclusions of our manuscript. There is no other way to monitor brain activity and provide a compelling proxy for amnesia/awareness in the context of an ablated righting reflex. With our basic and non-invasive recording, we tried to compare EEGs between the Dex based combinations and the conventional anesthetic agents. We stated our observation without making any conclusive statement on amnesia. We feel some readers may be interested in the EEG information for the Dex based anesthesia. Some evidence suggested EEG monitoring is helpful to prevent overdose of anesthetics in certain vulnerable populations (2, 3, references below). A recent review paper suggested that intraoperative EGG monitoring is helpful to enhance recovery although there is insufficient outcome data (see reference 4 below). In our study, we compare the EEG spectrograms and power spectra with ~ 1 MAC of volatile agent vs Dex combinations and shown that the Burst-Suppression Ratio under ~ 1 MAC of volatile agent vs Dex combinations were very different.

We have shortened the EEG discussion by moving some statistical information to the figure legends. As the reviewer correctly points out, EEG data is complicated. As such we feel compelled to ensure we have provided sufficient information to the readers in the methods and analysis. We ask for the reviewer’s understanding.

***7. On Page 111 Lines 706-717, the authors concisely summarize their work and results. However, this is buried in the discussion. Similarly, the section on EEG effects (Page 112 Lines 721-738 is summarized nicely. This style of writing is appreciated for its ease of readability. The authors should look for other sections that could similarly be reduced.***

Thank you for your suggestion. We revised the discussion section accordingly.

***8. The figure legends often contain extremely long descriptions of the statistics derived from their work. It isn’t necessary and is impossible to read. If the authors feel this data is needed, then it should be in a supplemental figure/table or somehow included in the figure itself. For example, Figure 5 on Page 121 Line 928-961 is impossible to read. Other figure legends are similar.***

Thank you for the suggestion. We agree the extensive statistical description is not necessary and may cause confusion. We have re-written the figure 3, 5 and 7 legends and made them shorter. We used the number at the point before the application of Dex or Dex based combination as the baseline. We compared the rest of the time points to the baseline. We hope it is easier to read.

***9. Overall, a great revision but still central message is lost by the length and unnecessary detail included in the manuscript. The story is now clearer but the writing isn’t.***

***a) Is it possible to use dex as a primary agent (i.e., a higher dose)?
b) Can we overcome limitations of using higher dex doses (no reversal, slow emergence)?
c) Can co-administration of higher doses of dex further lower the amounts of other general anesthetic agents utilized (e.g., sevo or prop) as suggested by prior work?
d) If so, do such combinations of high dex with sevo or prop produce the other aspects of a general anesthetic (i.e., ability to perform surgery, effects on memory/amnesia)?***

We appreciate these suggestions and include all of them in the discussion.

**Reviewer #2: (No Response)**

Thank you for reviewing our MS.

**References:**

1. Khan ZP, Munday IT, Jones RM, Thornton C, Mant TG, Amin D. Effects of dexmedetomidine on isoflurane requirements in healthy volunteers.1: Pharmacodynamic and pharmacokinetic interactions Br J Anaesth. 1999; 83:372–80
2. Koch S et al. Perioperative electroencephalogram spectral dynamics related to postoperative delirium in older patients, Anesthesia & Analg. 2021
3. Yuan I et al. Prevalence of isoelectric electroencephalography events in infants and young children undergoing general anesthesia, Anesthesia & Analg. 2021
4. Chan, MTV et al. American Society for Enhanced Recovery and Perioperative Quality Initiative Joint Consensus Statement on the Role of Neuromonitoring in Perioperative Outcomes: Electroencephalography, Anesthesia & Analg. 2020

Respectful

Zheng (Jimmy) Xie, MD, Ph.D, FASA

Professor

Department of Anesthesia and Critical Care

University of Chicago
